# Supplementary material for: Organic additive release from plastic to seawater is lower under deep-sea conditions
Source: Nat Commun. 2021 Jul 20;12:4426. doi: 10.1038/s41467-021-24738-w (PMC8292457; doi:10.1038/s41467-021-24738-w)
Supplement: Supplementary file 1 — Supplementary Information [file 41467_2021_24738_MOESM1_ESM.pdf]

Organic additive release from plastic to seawater is lower under deep-sea conditions

Supplementary information

Authors: Vincent Fauvelle[1,*], Marc Garel[1], Christian Tamburini[1], David Nerini[1], Javier Castro-Jiménez[2], Natascha Schmidt[1], Andrea Paluselli[3], Armand Fahs[4], Laure Papillon[1], Andy M. Booth[5], Richard Sempéré[1]

[1]Aix-Marseille Univ., Toulon Univ., CNRS, IRD, Mediterranean Institute of Oceanography (MIO), UM 110, Marseille, France

[2]IFREMER, Laboratory of Biogeochemistry of Organic Contaminants (LBCO), Rue de l'Ile d'Yeu, BP 21105, 44311, Nantes, Cedex 3, France

[3]Incheon National University, Department of Basic Science, South Korea

[4]University of Toulon, Laboratory MAPIEM (EA 4323), CS 60584, 83041 Toulon Cedex 9, France

[5]SINTEF Ocean AS, Environment and New Resources, Trondheim NO-7465, Norway

*Corresponding author: Vincent Fauvelle, vincent.fauvelle@ird.fr

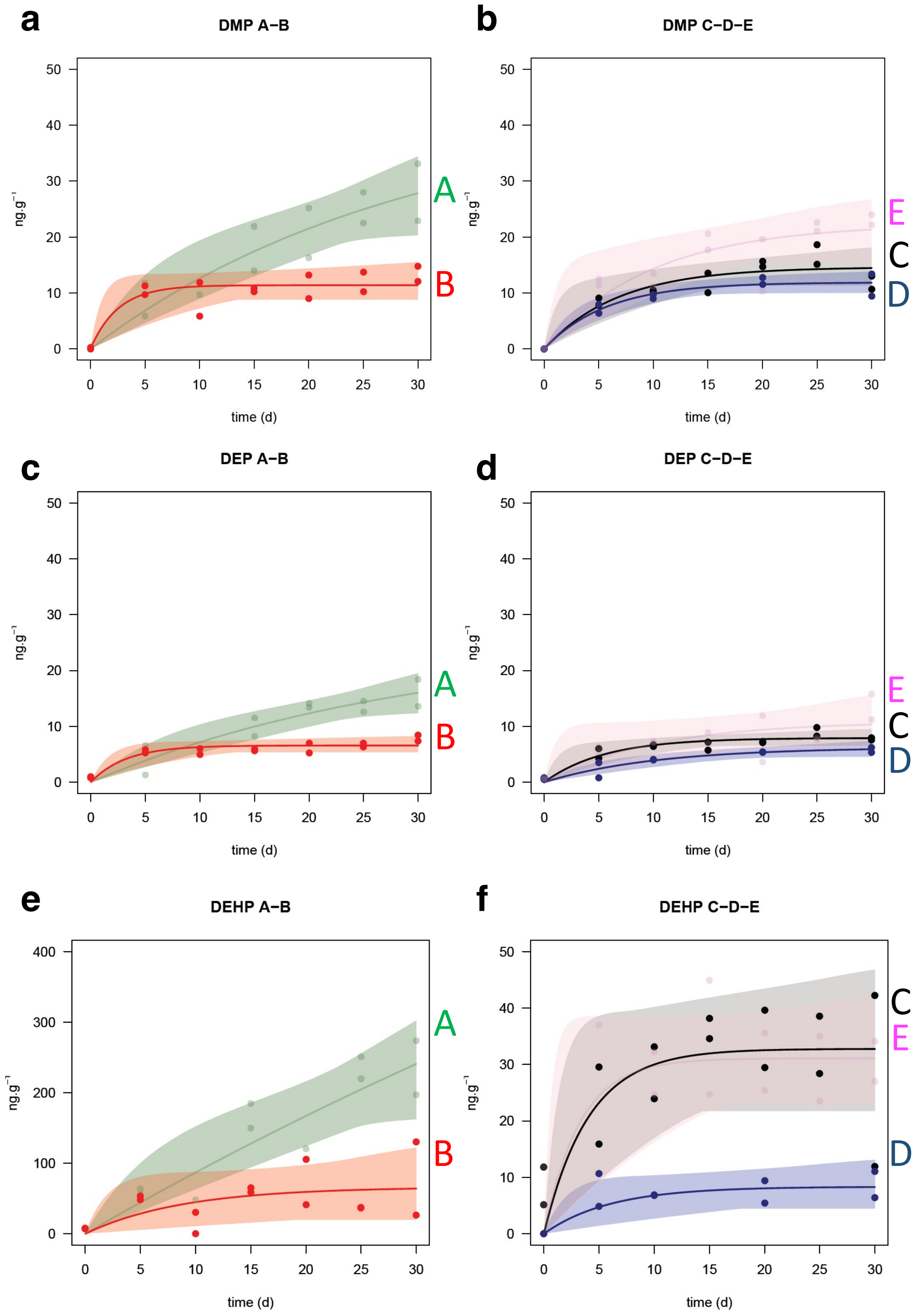
a
DMP A−B
b
DMP C−D−E
c
DEP A−B
d
DEP C−D−E
e
DEHP A−B
f
DEHP C−D−E
ng.g⁻¹
time (d)
A
B
C
D
E

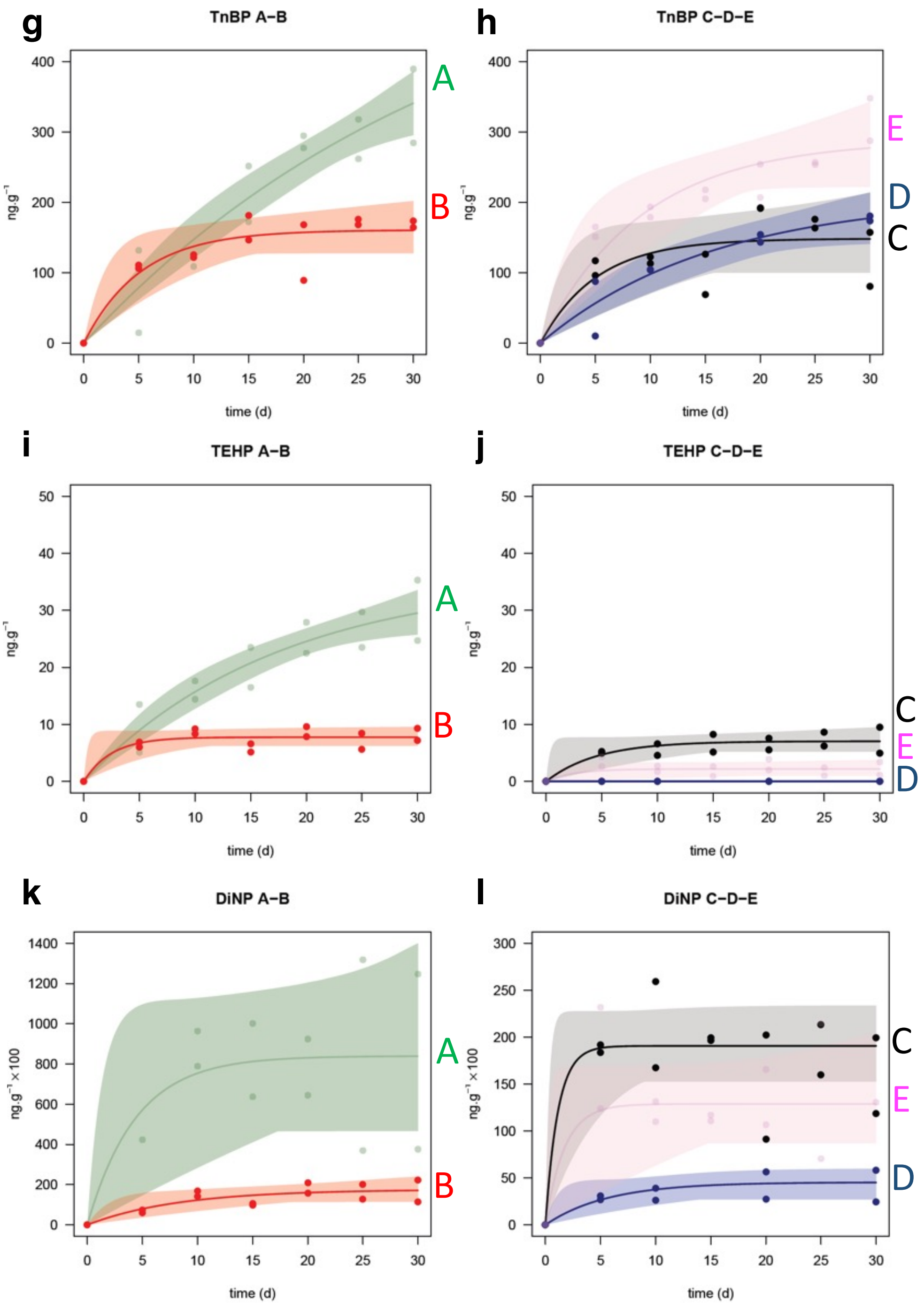

Supplementary Figure 1. Release of plastic additives from polyethylene (dimethyl phthalate DMP panel **a** and **b**, diethyl phthalate DEP panels **c** and **d**, diethylhexyl phthalate DEHP panels **e** and **f**, tri-n-butyl phosphate TnBP panels **g** and **h**, tris(2-ethylhexyl) phosphate TEHP panels **i** and **j**) and PVC (diisononyl phthalate DiNP panels **k** and **l**) upon a 30-days exposure to 0.1 and 10 MPa under biotic and abiotic conditions and in water originating from surface or deep-sea environments. Error bands represent the 95% confidence intervals.

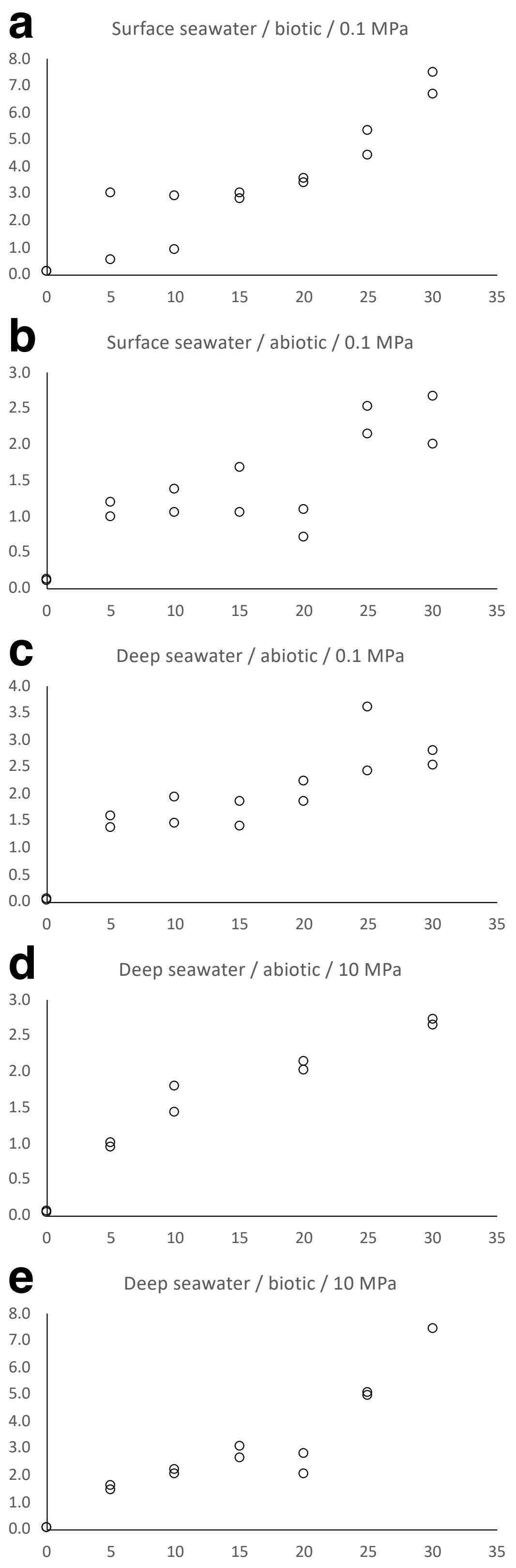

Supplementary Figure 2. Bisphenol S release from polyvinyl chloride (ng g^{-1}) upon a 30-day exposure under varying conditions of seawater quality (surface seawater panels **a** and **b**, deep seawater panels **c**, **d**, **e**), prokaryote presence (biotic/abiotic) and pressure (0.1 and 10 MPa).

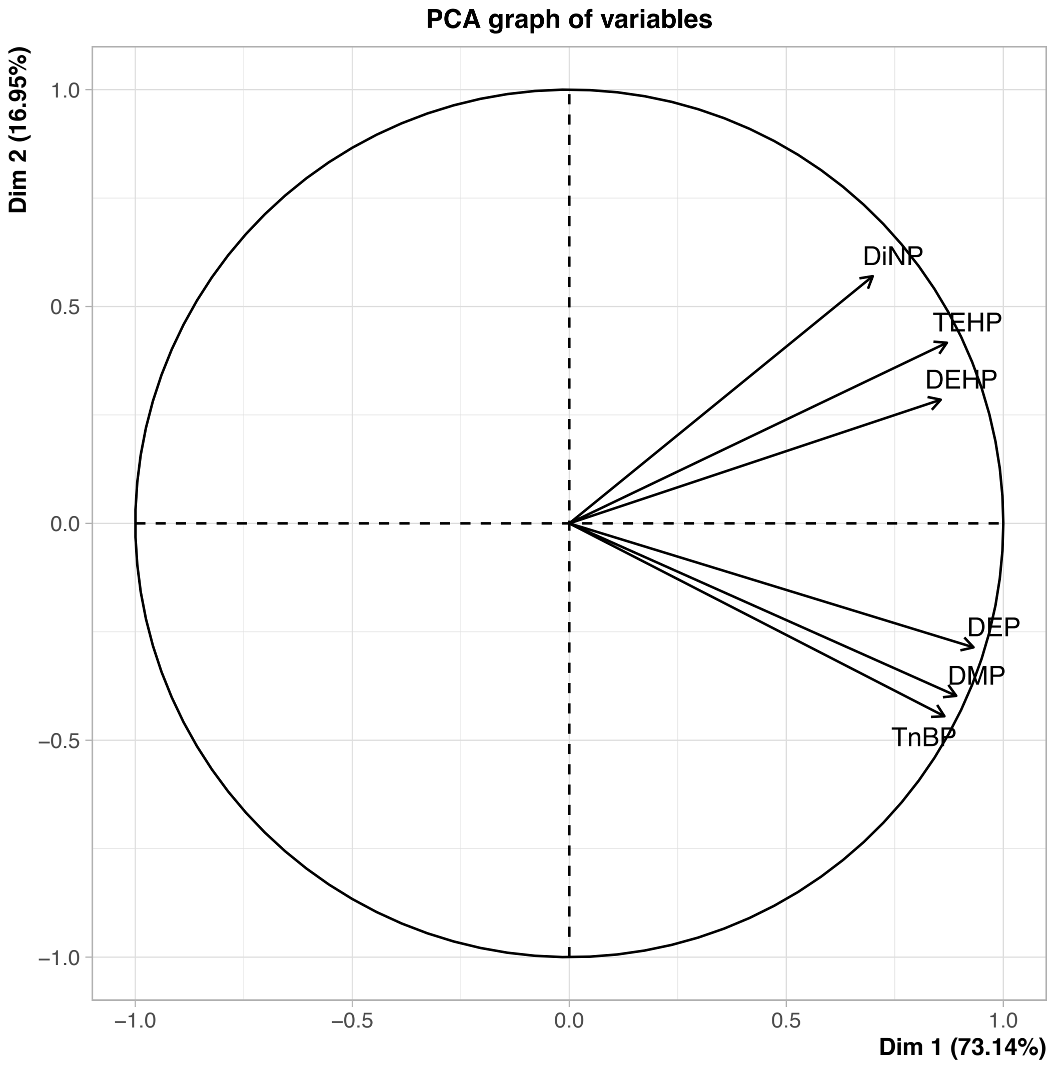

Supplementary Figure 3. Principal Component Analysis (PCA) performed an all additives release data.

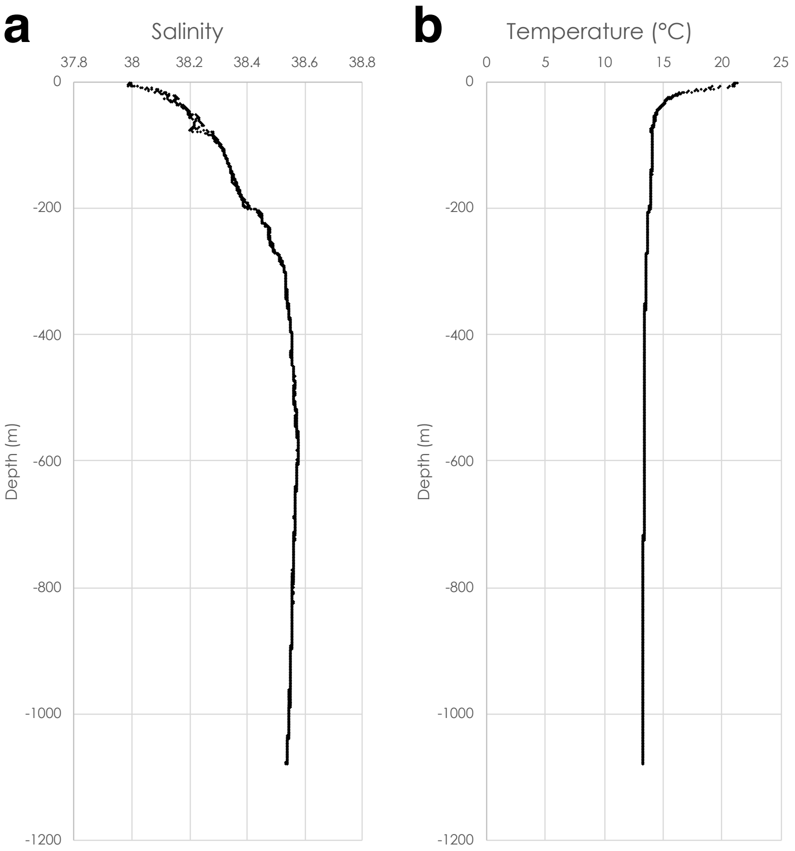

Supplementary Figure 4. Salinity (panel **a**) and temperature (panel **b**) profiles at the deep seawater sampling site (43.068223 °N, 5.468057 °E, 15 km from Marseille coastline) made at 1000 m depth the 6th june 2018.

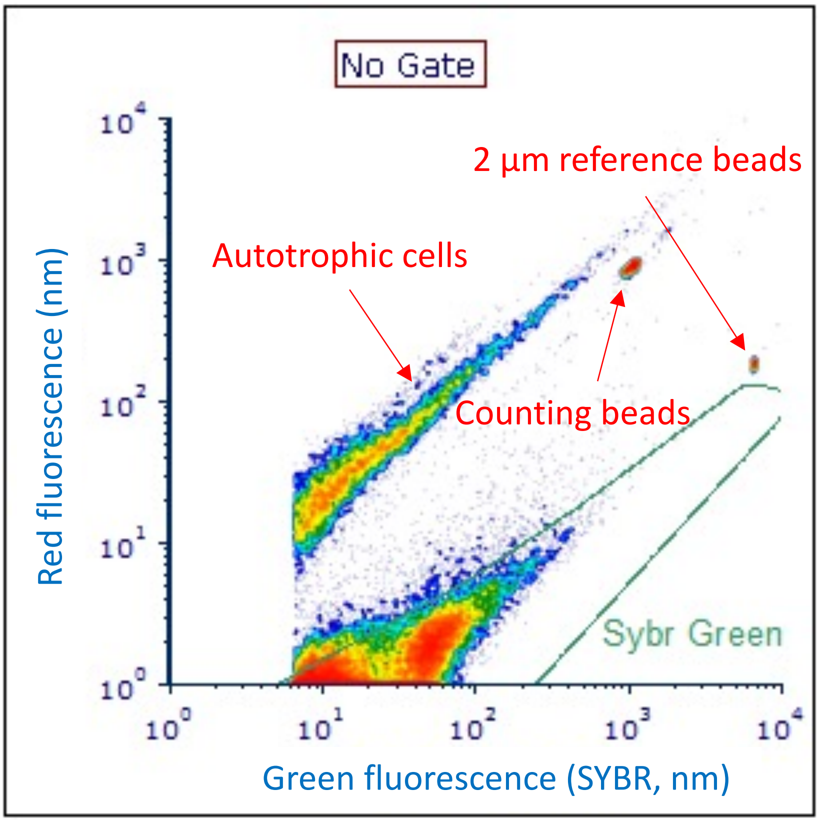

Supplementary Figure 5. Gating and sorting strategy for flow cytometry data. Reference beads are Fluoresbrite™ (Polysciences). Counting beads are Trucount™ (BDBiosciences). Elimination of autotrophic cells and identification of SYBR Green positive cells based on i) red fluorescence of chlorophyll a (detection via a 670LP filter, removing signals with wavelengths above 670nm), and ii) the green fluorescence due to SYBR Green (detection via a 530/30BP filter capturing signals with wavelengths between 515 and 545nm).
