# Supplementary material for: Organic additive release from plastic to seawater is lower under deep-sea conditions
Source: Nat Commun. 2021 Jul 20;12:4426. doi: 10.1038/s41467-021-24738-w (PMC8292457; doi:10.1038/s41467-021-24738-w)
Supplement: Supplementary file 3 — Reporting summary [file 41467_2021_24738_MOESM3_ESM.pdf]

nature portfolio

Corresponding author(s): Vincent Fauvelle

Last updated by author(s): Jun 16, 2021

Reporting Summary

Nature Portfolio wishes to improve the reproducibility of the work that we publish. This form provides structure for consistency and transparency in reporting. For further information on Nature Portfolio policies, see our Editorial Policies and the Editorial Policy Checklist.

Statistics

For all statistical analyses, confirm that the following items are present in the figure legend, table legend, main text, or Methods section.

n/a	Confirmed	
☐	☒	The exact sample size (n) for each experimental group/condition, given as a discrete number and unit of measurement
☐	☒	A statement on whether measurements were taken from distinct samples or whether the same sample was measured repeatedly
☒	☐	The statistical test(s) used AND whether they are one- or two-sided<br>*Only common tests should be described solely by name; describe more complex techniques in the Methods section.*
☐	☒	A description of all covariates tested
☐	☒	A description of any assumptions or corrections, such as tests of normality and adjustment for multiple comparisons
☐	☒	A full description of the statistical parameters including central tendency (e.g. means) or other basic estimates (e.g. regression coefficient) AND variation (e.g. standard deviation) or associated estimates of uncertainty (e.g. confidence intervals)
☒	☐	For null hypothesis testing, the test statistic (e.g. F, t, r) with confidence intervals, effect sizes, degrees of freedom and P value noted<br>*Give P values as exact values whenever suitable.*
☒	☐	For Bayesian analysis, information on the choice of priors and Markov chain Monte Carlo settings
☐	☒	For hierarchical and complex designs, identification of the appropriate level for tests and full reporting of outcomes
☒	☐	Estimates of effect sizes (e.g. Cohen's d, Pearson's r), indicating how they were calculated

Our web collection on statistics for biologists contains articles on many of the points above.

Software and code

Policy information about availability of computer code

Data collection	No software used
Data analysis	No software used

For manuscripts utilizing custom algorithms or software that are central to the research but not yet described in published literature, software must be made available to editors and reviewers. We strongly encourage code deposition in a community repository (e.g. GitHub). See the Nature Portfolio guidelines for submitting code & software for further information.

Data

Policy information about availability of data

All manuscripts must include a data availability statement. This statement should provide the following information, where applicable:

- Accession codes, unique identifiers, or web links for publicly available datasets
- A description of any restrictions on data availability
- For clinical datasets or third party data, please ensure that the statement adheres to our policy

The authors declare that the data supporting the findings of this study are available within the article.

Field-specific reporting

Please select the one below that is the best fit for your research. If you are not sure, read the appropriate sections before making your selection.

☐ Life sciences ☐ Behavioural & social sciences ☒ Ecological, evolutionary & environmental sciences

For a reference copy of the document with all sections, see nature.com/documents/nr-reporting-summary-flat.pdf

Ecological, evolutionary & environmental sciences study design

All studies must disclose on these points even when the disclosure is negative.

Study description	Kinetic release of organic additives release from plastic under deep-sea conditions
Research sample	Polyehtylene and polyvinylchloride were chosen due to their high industrial production volume, and differences in behaviour (density higher than that of water for polyvinylchloride, lower thant that of water for polyethylene).
Sampling strategy	Independent duplicate samples were taken each 5 days during 1 month, as a preliminary study showed that additive release occurs during the first 2 weeks. The total amount of data allows fitting a first-order kinetic properly.
Data collection	Marc Garel and Vincent Fauvelle were present during data collection.
Timing and spatial scale	Surface and deep-seawater were sampled november 23rd 2018, and June 6th 2018 respectively. Following incubation were stopped when carbon, nitrogen and phosphorous release reached a plateau.
Data exclusions	No data excluded
Reproducibility	All attemps at replication were successfull
Randomization	Randomization was not relevant to our study, since all incubation parameters were controlled, and therefore grouped in that way.
Blinding	All data were aquired at the end of the experiment in a single batch, blinding was therefore not necessary/applicable.

Did the study involve field work? ☒ Yes ☐ No

Field work, collection and transport

Field conditions	Calm water, sunny, water temperature between 11 and 13 °C at the sampling location.
Location	43.068223 °N, 5.468057 °E, 1000 m depth; 43.273624 °N, 5.347348 °E, 0.5 m depth
Access & import/export	Sampling in agreement with the Calanques Noational Park, no permits needed for water sampling.
Disturbance	No disturbance

Reporting for specific materials, systems and methods

We require information from authors about some types of materials, experimental systems and methods used in many studies. Here, indicate whether each material, system or method listed is relevant to your study. If you are not sure if a list item applies to your research, read the appropriate section before selecting a response.

Materials & experimental systems

n/a	Involved in the study	
☒	☐	Antibodies
☒	☐	Eukaryotic cell lines
☒	☐	Palaeontology and archaeology
☒	☐	Animals and other organisms
☒	☐	Human research participants
☒	☐	Clinical data
☒	☐	Dual use research of concern

Methods

n/a	Involved in the study	
☒	☐	ChIP-seq
☐	☒	Flow cytometry
☒	☐	MRI-based neuroimaging

Flow Cytometry

Plots

Confirm that:

☒ The axis labels state the marker and fluorochrome used (e.g. CD4-FITC).

☒ The axis scales are clearly visible. Include numbers along axes only for bottom left plot of group (a 'group' is an analysis of identical markers).

☒ All plots are contour plots with outliers or pseudocolor plots.

☒ A numerical value for number of cells or percentage (with statistics) is provided.

Methodology

Sample preparation	Immediately after sampling, the samples were thawed at room temperature and stained using SYBR Green II (Molecular Probes®).
Instrument	FACSCalibur flow cytometer (BD Biosciences®) equipped with an air-cooled argon laser (488 nm) and a red diode (633 nm)
Software	CellQuestPro and FCS Express
Cell population abundance	100000 heterotrophic porkaryotes magnitude, from a natural surface and deep-seawater assemblages.
Gating strategy	Elimination of autotrophic cells and identification of SYBR Green positive cells based on :<br>- red fluorescence from chlorophyll a (detection via a 670LP filter, harvesting signals with wavelengths above 670nm)<br>- and the green fluorescence due to SYBR Green (detection via a 530/30BP filter collecting signals of wavelength between 515 and 545nm).

☒ Tick this box to confirm that a figure exemplifying the gating strategy is provided in the Supplementary Information.
